# De Novo *ESR1* Hotspot Mutation in a Patient With Endometrial Cancer Treated With an Aromatase Inhibitor

**DOI:** 10.1200/PO.18.00398

**Published:** 2019-05-21

**Authors:** Adeline Morel, Julien Masliah-Planchon, Guillaume Bataillon, Véronique Becette, Claire Morel, Samantha Antonio, Elodie Girard, Ivan Bièche, Christophe Le Tourneau, Maud Kamal

**Affiliations:** ^1^ Institut Curie, PSL Research University, Paris, France; ^2^ Institut Curie, PSL Research University, Paris & Saint-Cloud, France; ^3^ Institut Curie, Paris & Saint-Cloud, France; ^4^ INSERM U900, Institut Curie, Saint-Cloud, France; ^5^ INSERM U1016, Paris Descartes University, Paris, France; ^6^ Paris-Saclay University, Paris, France

## INTRODUCTION

Endometrial carcinoma is the fourth most frequent cancer in women. Endometrial carcinoma is classified into two subtypes: type 1 endometrial carcinoma characterized by estrogen receptor (ER) expression and obesity, and type 2 endometrial carcinoma in nonobese, older women. Type 1 endometrial carcinoma is associated with a favorable prognosis, whereas type 2 has a poorer prognosis.^1^ A prognostic genomic classification of endometrial cancers into four groups has been established using exome sequencing; however, this classification is not used in the clinic.^2^

Standard-of-care treatment of endometrial carcinoma consists of primary hysterectomy, bilateral salpingo-oophorectomy, and pelvic lymph node dissection followed by adjuvant therapy based on the histologic assessment of the specimen.^3^ Chemotherapy is proposed in the recurrent and/or metastatic setting, whereas hormone therapy represents a treatment option for patients with ER expression.^4^ In breast cancer, *ESR1* mutations were clearly identified as a mechanism of resistance to aromatase inhibitors.^5-8^ *ESR1* mutations were reported in 2% of endometrial cancers,^9^ yet their potential occurrence following hormonal therapy is unknown. In this study, we report a de novo *ESR1* hotspot mutation in a patient with endometrial carcinoma treated with an aromatase inhibitor.

## CASE REPORT

A 56-year-old woman was diagnosed in December 2010 with a grade 1 endometrioid carcinoma on biopsy. The patient underwent a total hysterectomy with bilateral annexectomy, omentectomy, and pelvic and aortic lymph node dissection. The diagnosis of grade 1 endometrioid carcinoma was confirmed, with less than 50% invasion of the myometrial wall thickness, 4 cm in greatest dimension, lymphovascular invasion, bilateral ovarian metastases, and no pelvic lymph node metastases (pT3aN0 Fédération Internationale de Gynécologie et d’Obstétrique IIIA). Using immunohistochemistry, tumor cells were shown to express ER, progesterone receptor, and a wild-type staining of p53. In addition, tumor cells expressed CK7 and PAX8 and did not express CK20, TTF1, CDX2, and WT1, confirming the endometrial origin of the tumor.

**FIG 1.**
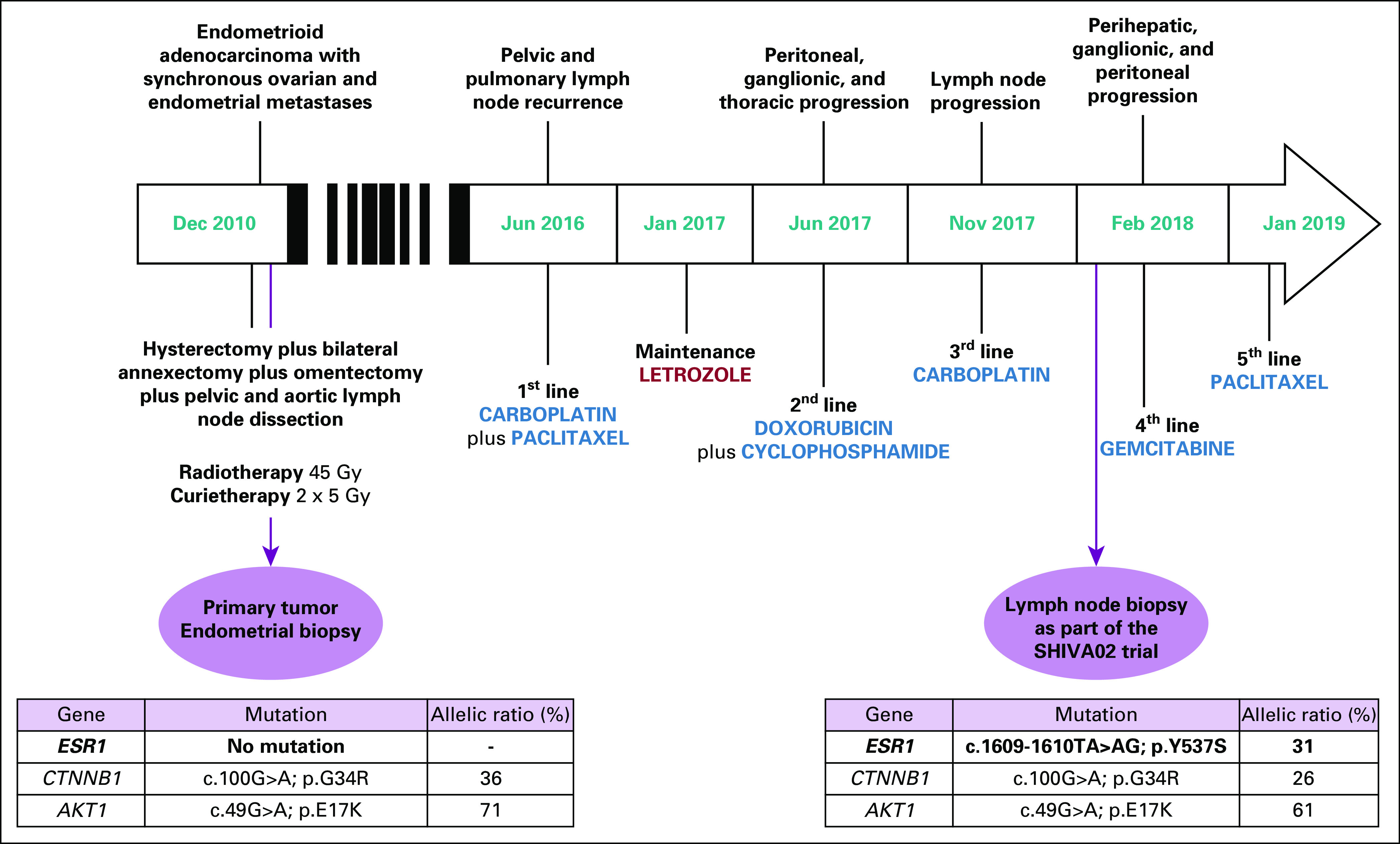
Treatment schedule and *ESR1* mutations. SHIVA02, Evaluation of the Efficacy of Targeted Therapy Based on Tumor Molecular Profiling in Patients With Advanced Cancer Using Each Patient as Its Own Control (ClinicalTrials.gov identifier: NCT03084757).

The patient then received adjuvant external pelvic radiotherapy at a dose of 45 Gy followed by two sessions of vaginal Curietherapy at a dose of 5 Gy. The patient had regular follow-up visits at Institut Curie until June 2016, when she had a pelvic recurrence that was confirmed histologically. The patient was treated with first-line chemotherapy with carboplatin and paclitaxel. After nine cycles of chemotherapy, the patient received maintenance therapy with an aromatase inhibitor (ie, letrozole). After receiving letrozole for 6 months, the patient experienced disease progression and was subsequently treated with doxorubicin and cyclophosphamide and then carboplatin alone. The patient was then biopsied in the framework of the SHIVA02 trial (Evaluation of the Efficacy of Targeted Therapy Based on Tumor Molecular Profiling in Patients With Advanced Cancer Using Each Patient as Its Own Control; ClinicalTrials.gov identifier: NCT03084757), which aimed to identify druggable molecular alterations, and received a fourth line of chemotherapy with gemcitabine (Fig 1).

Molecular profiling was performed on a frozen biopsy of a metastatic lymph node in the SHIVA02 trial using a dedicated targeted sequencing panel covering 80 genes. Targeted sequencing revealed an activating *ESR1* hotspot exon 8 mutation (c.1609-1610TA>AG; p.Y537S) reported in the COSMIC (Catalogue of Somatic Mutations in Cancer) database (COSM 6948665) with an allelic ratio of 31%. Other molecular alterations included an *AKT1* mutation (c.49G>A; p.E17K) with an allelic ratio of 61% and a *CTNNB1* mutation (c.100G>A; p.G34R) with an allelic ratio of 26%. ER and progesterone receptor were expressed in 30% and 100% of cells, respectively. No microsatellite instability was detected. No *ESR1* amplification was identified (Fig 1).

To assess the potential de novo character of the *ESR1* mutation, we analyzed the primary formalin-fixed paraffin-embedded endometrial tumor at diagnosis using targeted sequencing. No *ESR1* mutation was identified (with a sensitivity of detection of 1%). However, *AKT1* (c.49G>A; p.E17K) and *CTTNB1* (c.100G>A; p.G34R) mutations were observed in the primary tumor with 71% and 36% allelic ratios, respectively, which were comparable to mutations found in the distant metastasis (Fig 1).

## DISCUSSION

We identified a de novo *ESR1* mutation in our patient with endometrial carcinoma treated with an aromatase inhibitor. The activating *ESR1* hotspot exon 8 mutation (c.1609-1610TA>AG; p.Y537S) identified was previously reported in patients with breast cancer treated with the same hormone therapy.^5-8^ These data highlight the putative secondary resistance characteristic of the *ESR1* mutation detected in our case report.

In 1996, *ESR1* hotspot mutations were first described in cell models, where they were found to confer constitutive activation of the ER and secondary resistance to hormone therapy.^10^ *ESR1* gene amplifications, on the other hand, were not reported as a potential second mechanism of resistance to hormone therapy.^7^ *ESR1* mutations in the ligand-binding domain are activating and drive transcription and cell proliferation in the absence of estrogen. In patients with breast cancer, *ESR1* hotspot mutations in the ligand-binding domain were observed exclusively after hormone therapy^5-8^ and have been shown to be associated with poor prognosis.^11^ After exposure to aromatase inhibitors, the prevalence of *ESR1* mutations was significantly higher in patients exposed during the metastatic phase than during the adjuvant phase (36% *v* 6%).^11^ In a recent study, advanced breast cancer tumors harboring *ESR1* mutations, or alterations in genes involved in the mitogen-activated protein kinase pathway and in the ER transcriptional machinery, were shown to barely benefit from aromatase inhibitors.^5^

*ESR1* amplifications were detected in 12% of endometrial carcinomas,^12^ whereas *ESR1* hotspot mutations were less frequent (2%).^9^ *ESR1* amplifications were shown to be associated with a poor prognosis and seemed to be an early event in endometrial carcinoma development.^12^ Selective ER degraders, such as fulvestrant, were shown to be effective in patients with hormone receptor–positive breast cancer. *ESR1* mutations were not reported to be associated with clinical resistance to fulvestrant.^13^

To our knowledge, our case report is the first to report a potential de novo *ESR1* mutation in a patient with ER-positive endometrial carcinoma treated with an aromatase inhibitor. The de novo characteristic of the *ESR1* mutation should be considered in the context of multiple lines of systemic chemotherapy received between the initial biopsy and the second biopsy. The incidence and predictive value of the *ESR1* mutation have yet to be investigated in a large series of patients with endometrial carcinoma treated with aromatase inhibitors. The development of ESR1 inhibitors may be of interest in patients who develop this mutation resistance.
